# Transfer and Metabolism of Triadimefon Residues from Rape Flowers to Apicultural Products

**DOI:** 10.1155/2017/7697345

**Published:** 2017-09-06

**Authors:** Ying-Hong Li, Bei-Lei Zhou, Ming-Rong Qian, Qiang Wang, Hu Zhang

**Affiliations:** ^1^Zhejiang Institute for Food and Drug Control, Hangzhou 310052, China; ^2^Institute of Quality and Standard for Agricultural Products, Zhejiang Academy of Agricultural Sciences, Hangzhou 310021, China

## Abstract

This paper presents a study on the transfer and metabolism of triadimefon residues from rape flowers to apicultural products. In the field trials, honeybee colonies were placed in four rape greenhouses treated with triadimefon on standard dosage. Apicultural products (pollen, honey, and royal jelly) were collected on a regular basis. Sample preparation and extraction procedure were established. HPLC/ESI-MS/MS method was validated. The respective residues of triadimefon and metabolite triadimenol were 0.03 ± 0.002 mg/kg and 0.13 ± 0.02 mg/kg in pollen on the 18th day, and both had reached the limits of detection in honey on the 24th day, while they were 0.004 ± 0.0005 mg/kg and 0.010 ± 0.0002 mg/kg in royal jelly on the 22nd day. Mathematical curve fitting studies were further investigated. On the basis of recommended dosage, the degradation half-lives of triadimefon in pollen, honey, and royal jelly were about 0.7, 12.5, and 19.5 days, respectively. Transfer of triadimefon residues from rape flowers to apicultural products diminished over spraying time. The residues of triadimefon and metabolite triadimenol in pollen were about 10 times higher than those in honey and jelly. Time to attain the maximum permissible limit of pollen in the European Union was 14.9 days, predicted from the index function.

## 1. Introduction

Bees are important natural pollinators not only for crops but also for wild flowers and plants in natural ecosystems [1, 2]. The widespread use of pesticides has caused pollution in bees [3, 4]. As a direct result, bees have become important spreaders of pesticides [5]. Apicultural products extracted from bees such as pollen, honey, and jelly are popular agricultural products worldwide. The quality of apicultural products is dependent on whether forage plants are contaminated by pesticides. In China, rape is widely planted in the Yangtze River Basin due to the high nutritional and economic value of rapeseed. In addition, rape is the main nectar source for bees in the region. However, rape is susceptible to insect pests especially fungal diseases during its entire growth period, which can significantly impact yield [6]. While spraying with insecticides and fungicides protects the crop [7], it conversely leads to the contamination of apicultural products. A wide range of neonicotinoid insecticides and fungicides have been detected in pollen produced by bees fluttering among oilseed rape and wild flowers nearby and reveal the serious consequences of simultaneous exposure of bees to complex mixtures of pesticides [8]. Chinese annual output and exports of apicultural products have ranked first in the world for many years. Several countries, such as the European Union and Japan, have raised technical trade barriers on exports of Chinese apicultural products due to the high levels of chemical pesticide residues [9]. Therefore, it is of great significance to carry out studies on the transfer and metabolism of pesticide residues from forage plants to apicultural products.

Pesticide residues in apicultural products have been researched for many years. The multiresidue method for the determination of pesticides and pesticide metabolites in honeybees has been developed and validated [10]. Several studies have investigated the transfer rates and proportions of pesticide residues, such as transfer assessment of fipronil residues from feed to cow milk [11], pesticide residue transfer rates (percent) from dried tea leaves to brewed tea [12, 13], chlorpyrifos residual behaviors in field crops and transfer during duck pellet feed processing [14], and chlorpyrifos transferred from contaminated feed to duck commodities and dietary risks [15]. However, experimental studies on pesticide accumulation from nectar plants to apicultural products are not enough in depth [16, 17]. Diazinon and thiacloprid residues in pollen were determined after orchard treatment, and possible sublethal effects on individual honeybees and brood were discussed [18]. Concentration of bifenthrin, fenpyroximate, methidathion, spinosad, thiamethoxam, and triazophos exceeded the maximum residue levels in several honey samples, and the kinds of the residues were correlated to agriculture practices in Northern Poland [19]. In summary, it is necessary and urgent to apply different approaches to monitor fluctuations of transferred residues after pesticide exposure in field trials.

As State Key Laboratory for Pesticide Residue Detection of the Ministry of Agriculture, we are accumulating experiences in agriculture and food safety and attempting to build up entire proceeding research systems from the farmland to the dining table [14, 15, 20–22]. We have attempted to provide a comprehensive risk assessment for different pesticide residues found in pollen, honey, and royal jelly. We have validated preparation and extraction procedures in the laboratory and designed field trials to investigate the transfer assessment of carbendazim residues from rape flowers to apicultural products [20]. Carbendazim is a benzimidazole fungicide, while triadimefon is a chloroarene fungicide used in agriculture to control various fungal diseases. It is particularly worth mentioning that triadimefon and its metabolite triadimenol have been simultaneously detected in edible vegetable oil by our former research [21]. Previous studies have shown that triadimefon and its metabolite triadimenol exhibit clear teratogenic effects and are harmful to the mammalian central nervous system [23]. The regulatory level of triadimefon and triadimenol quoted from Commission Regulation (EC) number 396/2005 of the European Parliament and of the council was 0.1 mg/kg.

This research aimed to validate the different procedures and detect the regulatory levels of pesticide residues and then open our minds to detect, recognize, and grasp the regularity of transfer and metabolism and finally to improve the security level of apicultural products. To achieve this objective, the present work was designed as follows. Rape flowers were sprayed with triadimefon during the flowering period in field trials. Pollen, honey, and royal jelly trapped by bees flying over the sprayed rape were collected at specific intervals. Transfer and metabolism of triadimefon residues from rape flowers to apicultural products were investigated from regulatory levels determined by the validated HPLC/ESI-MS/MS method. The degradation half-lives of triadimefon in pollen, honey, and royal jelly were obtained by fitting a curve to the exponential function. Time to attain the maximum permissible limit of pollen in the European Union was predicted further. The obtained results will provide basis data to master safe intervals of corresponding fungicides, which is very important to improve the quality of our agricultural products and obtain the competitive advantage to break the trade barriers.

## 2. Materials and Methods

### 2.1. Materials and Chemicals

Methanol (99.9% purity) and acetonitrile (99.9% purity) were procured from Merck (Darmstadt, Germany). Acetic acid (99% purity) was obtained from Sigma-Aldrich Co. (St. Louis, USA). The Oasis HLB cartridges (6 cc/150 mg, 30 *μ*m) were purchased from Waters Ltd. (Milford, USA). QuEChERS Extraction kits (1.5 g anhydrous sodium acetate and 6 g anhydrous MgSO_4_, 50 mL centrifuge tubes) and QuEChERS Clean-Up kits (150 mg anhydrous MgSO_4_, 50 mg Primary and Secondary Amine (PSA), and 50 mg C_18_, 2 mL centrifuge tubes) were obtained from Phenomenex Inc. (California, USA). Water (18.2 MΩ·Cm) was purified by Barnstead Nanopure System from Thermo Fisher Scientific Inc. (Massachusetts, USA). All other chemicals were of analytical reagent grade.

25% triadimefon wettable powder was provided by Zhongxun Agri-Science Co. (Guangdong, China). Pesticide standards triadimefon (99.0% purity) and triadimenol (99.0% purity) were purchased from Dr. Ehrenstorfer GmbH (Augsburg, Germany). By dissolving the reference compounds in methanol, stock standards (approximately 20 mg/L) of triadimefon and triadimenol were prepared. Working standards with lower concentrations were obtained by serial dilution of the stock standards with blank matrixes.

### 2.2. Field Trials

Field trials were designed the same as our former studies [20]. Under organic farming standards, rape of Zheshuang 72 double low* Brassica* was cultivated averagely in four GP 622 single-span greenhouses numbered from P_1_ to P_4_. P_1_ was blank control group while P_2_ to P_4_ were parallel experimental groups. During the flowering period, 25% triadimefon wettable powder was diluted with water and applied directly to rape in P_2_ to P_4_ greenhouses. The spraying concentration of triadimefon was 216 mg/kg as the recommended max concentration (127.5 g.a.i./ha). After pesticide spraying, 4 beehives containing approximately 5000 bees in each hive were located in P_1_ to P_4_ greenhouses, respectively. Pollen, honey, and royal jelly produced from P_1_ to P_4_ greenhouses were collected regularly as shown in Table 1. All labeled apicultural products were carefully transported and immediately stored in the laboratory at −20°C until analyzed.

### 2.3. Preparation and Extraction Procedure

Preparation and extraction procedures for residues of triadimefon and triadimenol from apicultural products were almost the same as our former studies [20], with the exception of using isotopic labeling with heavy-labeled compound. The final extracts were filtered by 0.22 *μ*m membrane and then determined by HPLC/ESI-MS/MS.

### 2.4. HPLC/ESI-MS/MS Analysis

**Figure 1:**
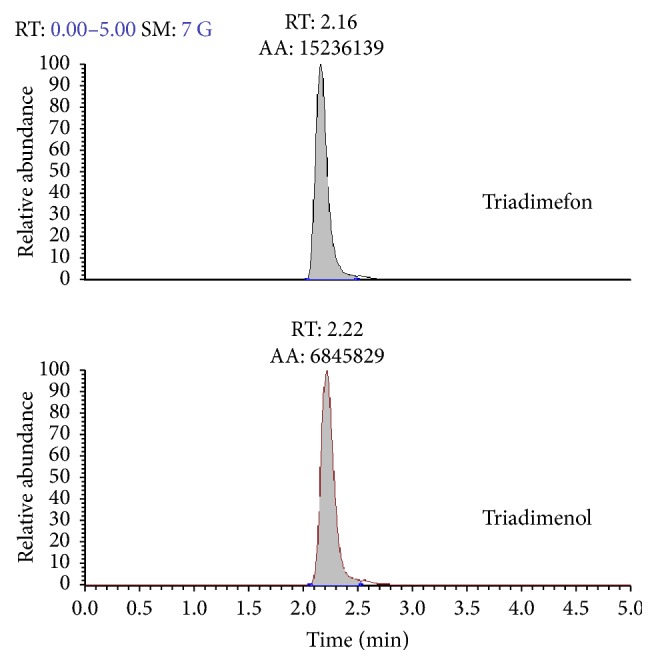
HPLC/ESI-MS/MS chromatograms of triadimefon and triadimenol in the standard solution.

The utilized HPLC/ESI-MS/MS system was a TSQ Discovery triple-quadrupole mass spectrometer coupled with a Surveyor liquid chromatograph from Thermo Fisher Scientific Inc. Quantitative data obtained from calibration standards and samples were processed by Xcalibur 2.0.7 software. HPLC conditions and ESI source conditions were coincident with our former studies [20].  Table 2 showed the MS/MS transitions selected for quantification and confirmation together with the optimized parameters for triadimefon and triadimenol. The retention time of triadimefon and triadimenol was approximately 2.16 and 2.22 min, respectively, as shown in Figure 1.

### 2.5. Transfer and Metabolism Studies

The transfer and metabolism of triadimefon residues from rape flowers to apicultural products were figured out by the HPLC/ESI-MS/MS method. Mathematical curve fitting was identified by a computer on the basis of transfer data.

In addition, the degradation half-lives of triadimefon in pollen, honey, and royal jelly were obtained from curve fitting by the exponential function. Time to attain the standard limit value of apicultural products in the European Union was predicted from the index function.

## 3. Results and Discussion

### 3.1. Optimization of Sample Pretreatment

Due to different physical properties such as consistency and fluidity, different methods were employed to pretreat apicultural products. The pretreatment method for pollen was developed from a fast and easy multiresidue method employing acetonitrile extraction/partitioning and dispersive solid-phase extraction for the determination of pesticide residues [24–27]. The pretreatment method for honey was improved from a confirmative method for sulfonamides, trimethoprim, and dapsone in honey by acidic hydrolysis and SPE [28]. The pretreatment method for royal jelly was improved from analysis of tetracycline residues in royal jelly by liquid chromatography-tandem mass spectrometry [29]. All pretreatments were modified and consisted of three steps. Firstly, target pesticides were extracted by suitable organic solvents. Extracts were cleaned up by dispersive solid-phase extraction technique in the second step. The third step comprised concentration, reconstitution, and filtration. The pretreatments had proven simple and operative in our laboratory [20].

### 3.2. Partial Validation of Analysis Method

Partial validation was carried out to evaluate the quantitative analysis method for triadimefon and triadimenol under the optimized HPLC/ESI-MS/MS conditions based on retention time and instrument resolution. Linear equation, linear range, accuracy, precision, limits of detection (LOD), and limits of quantification (LOQ) of triadimefon and triadimenol were determined, respectively, to validate the methods under the international guideline of ICH. Matrix effects could either enhance or suppress ionization of pesticide during the electrospray process [30]. Considering that the same pesticide presented different matrix effects, and in the same substrate, the matrix effects of tested pesticides were different from one another, matrix blank samples of apicultural products (pesticide-free) were chosen as reference matrixes, and matrix-matching calibration standards were prepared [31]. Matrix extracts were obtained according to the procedures mentioned in Section 2.3. In addition, the concentration range of the linear equation was chosen according to accuracy and sensitivity in SRM pattern of MS/MS analyses. Working standards at different concentrations were prepared by transferring 0.05, 0.25, 0.5, 2.0, 5.0, and 25.0 mL of 2.0 mg/L intermediate working solution into separate 100 mL volumetric flasks and making up to volume with blank matrix extracts in methanol. Calibration curves were constructed by concentration (mg/L) versus peak area in the form of *y* = *ax* + *b*. Parameters such as precision, accuracy, LOD, and LOQ were coincident with our former studies [20]. Tables 3 and 4 show validation parameters for triadimefon and triadimenol added in different blank samples and determined using matrix-matching calibration standards. The results illustrated the reliability and sensitivity to determine triadimefon and triadimenol in apicultural products.

### 3.3. Transfer and Metabolism of Triadimefon

**Figure 2:**
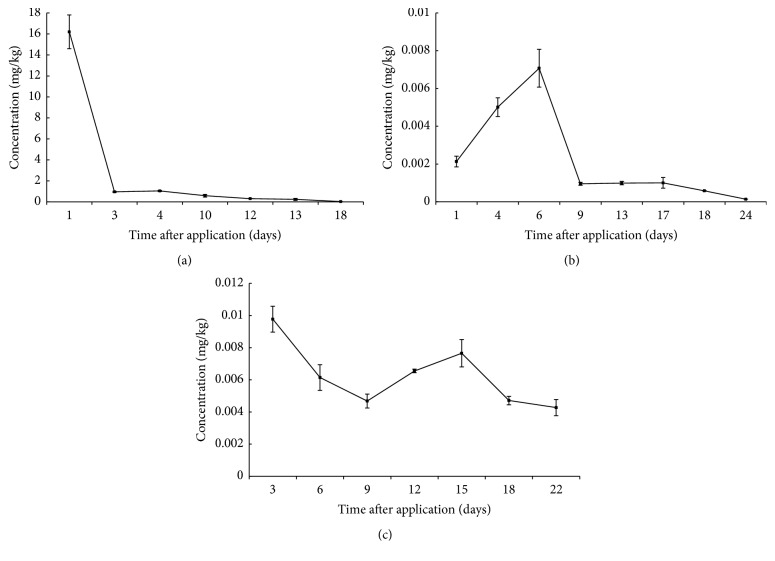
The concentration changes of triadimefon in apicultural products: (a) pollen, (b) honey, and (c) jelly.

**Figure 3:**
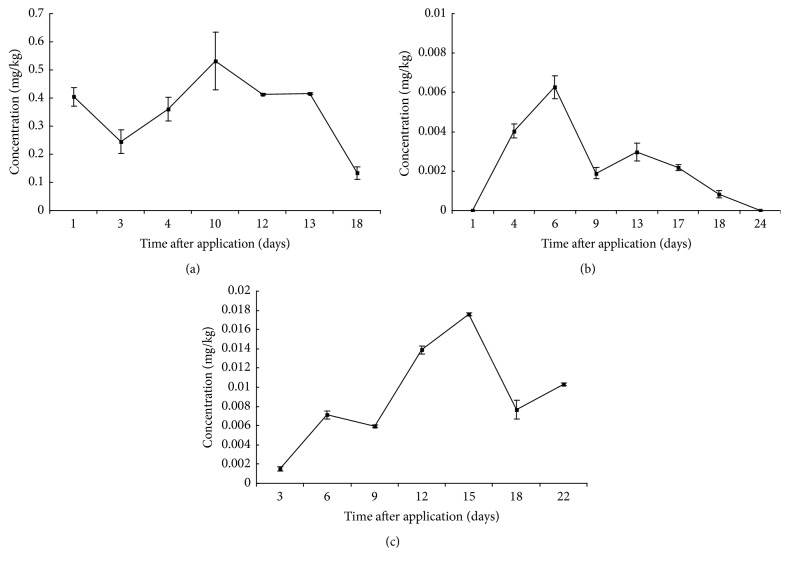
The concentration changes of metabolite triadimenol in apicultural products: (a) pollen, (b) honey, and (c) jelly.

The transfer and metabolism of triadimefon residues from rape flowers to apicultural products were calculated by HPLC/ESI-MS/MS method. Triadimefon and its metabolite triadimenol were detected in apicultural products. The variation of triadimefon is shown in Figure 2, while variation of metabolite triadimenol is shown in Figure 3. The residues of triadimefon and metabolite triadimenol in pollen were about 10 times higher than those in honey and jelly.

The concentrations of triadimefon and metabolite triadimenol in pollen were 16.2 ± 1.6 mg/kg and 0.40 ± 0.03 mg/kg 1 day after spraying, while they were 0.96 ± 0.06 mg/kg and 0.24 ± 0.04 mg/kg 3 days after spraying. That might be due to the rainy weather on the 2nd day after spraying. The rain might wash away the pesticide covering the surface of pollen. The data of the 4th day was higher than of the 3rd day, which might be due to uneven application. The residues of triadimefon showed a downward trend and declined at a slower rate 4 days later, while the residues of metabolite triadimenol almost kept the same level after 4 days later. After 12 days, the concentration of triadimenol became higher than that of triadimefon. The residues of triadimefon and metabolite triadimenol were at 0.03 ± 0.002 mg/kg and 0.13 ± 0.02 mg/kg on the 18th day.

Concentrations of triadimefon and metabolite triadimenol in honey were the highest at 6 days after spraying and then reduced. From 6 days to 24 days after spraying, the degradation rate of triadimefon became slower. Concentrations of triadimefon and metabolite triadimenol almost kept the same level during the period of 9 days and 17 days after spraying, while it suddenly turned out to be fast from 17 days to 24 days after spraying. After 9 days, the concentration of triadimenol became higher than that of triadimefon and both reached the LOD (0.0001 mg/kg) on the 24th day.

Concentration of triadimefon in jelly was the highest at 0.01 ± 0.001 mg/kg 3 days after spraying and then varied with time, while concentration of metabolite triadimenol reached the highest level at 0.018 ± 0.0001 mg/kg 15 days after spraying. After 6 days, the concentration of triadimenol became higher than that of triadimefon. The residue of triadimefon and metabolite triadimenol on the 22nd day was at 0.004 ± 0.0005 mg/kg and 0.010 ± 0.0002 mg/kg.

### 3.4. Mathematical Analysis for Curve Fitting

Considering that concentrations of triadimefon in apicultural products were diminished with time in general, the discrete experimental data to show the variation of triadimefon could be precisely fitted to mathematical curves. On the basis of transfer assessment of triadimefon from rape flowers to apicultural products, mathematical analysis for curve fitting was carried out further.

With the help of professional function drawing software including Excel (version 2013) and Origin 9.1, the fitting curves to investigate the degradation trends of triadimefon in apicultural products were obtained. Firstly, *P* value was obtained from Origin 9.1. The curve was fit if *P* < 0.05 [32]. Pesticide residues of corresponding time could be found from the drawn fitting curve [33]. Calibration curves were constructed as exponential functions, in the form of time after spraying pesticide application (days) versus triadimefon residues (mg/kg). By mathematical curve fitting, the exponential function simulation of triadimefon residues in pollen was *y* = 6.62*e*^−0.28*x*^ (*r* = 0.9145). *P* value was 1.95*∗*10^−6^ < 0.05, and therefore the reliability of curve fitting was confirmed. The degradation half-life of triadimefon in pollen was about 0.7 days which was obtained from curve fitting in the form of exponential function.

In this same way, the exponential function simulation of triadimefon residues in honey was *y* = 0.0055*e*^−0.13*x*^ (*r* = 0.8353). *P* value was 0.005 < 0.05. According to the recommended dose of triadimefon, the degradation half-life of triadimefon in honey was about 12.5 days according to the results obtained from curve fitting by exponential function.

By the same method, the exponential function simulation of triadimefon residues in royal jelly was *y* = 0.0086*e*^−0.029*x*^ (*r* = 0.7944). *P* value was 7.13*∗*10^−4^ < 0.05. The degradation half-life of triadimefon in jelly was about 19.5 days, calculated from curve fitting by exponential function.

### 3.5. Risk Assessment

The maximum permissible limit of triadimefon in apiculture products was 0.1 mg/kg in the European Union. According to the variation of triadimefon in apicultural products, concentrations of triadimefon detected in honey and royal jelly were always under the maximum permissible limit from the first sampling point to the end. But for pollen, the time required to attain the maximum permissible limit in the European Union was 14.9 days. The basis data and risk assessment could help farmers to keep safe intervals of corresponding fungicides. Also, the proposed field trial provided a scientific and reliable means for the risk assessment of pesticide residues from forage plants to apicultural products.

## 4. Conclusion

The results of this study indicate a scientific and reliable approach to study transfer and metabolism of pesticide residues from rape flowers to apicultural products by the HPLC/ESI-MS/MS method. The method is validated to be reliable and sensitive to determine triadimefon and metabolite triadimenol in apicultural products. Transfer and metabolism assessments of triadimefon residues from rape flowers to apicultural products show that the residues of triadimefon and metabolite triadimenol are varied with spraying time. The residues of triadimefon and metabolite triadimenol in pollen are about 10 times higher than those in honey and jelly. On the basis of the recommended dosage, the degradation half-lives of triadimefon in pollen, honey, and royal jelly are about 0.7, 12.5, and 19.5 days, respectively, according to the results obtained from curve fitting by exponential function. Time to attain the maximum permissible limit of pollen in the European Union is 14.9 days, predicted from the index function.

The study can not only help farmers to keep safe intervals of spraying corresponding fungicides, but also provide data support for the analysis of the residue limits and risk assessment of triadimefon in apicultural products and finally improve security level of apicultural products.

## Figures and Tables

**Table 1:** Actual sampling time after spraying application in field trials.

Apicultural products	Actual sampling time (days)							
Pollen	1	3	4	10	12	13	18	/
Honey	1	4	6	9	13	17	18	24
Jelly	3	6	9	12	15	18	22	/

**Table 2:** SRM conditions for target compounds.

Compounds	Parent mass (*m/z*)	Product mass (*m/z*)	Collision energy (V)
Triadimefon	294	197^a^	20
		69	17
Triadimenol	296	70^a^	30
		99	21

^a^Quantitative ion.

**Table 3:** Partial validation parameters of triadimefon added in different blank samples.

Parameters		Pollen	Honey	Jelly
Linear equation		*y* = 2*∗*10^7^*x* + 22896	*y* = 0.2*∗*10^7^*x* + 65306	*y* = 3.1*∗*10^7^*x* − 12944
Linear range (mg/L)		0.001–0.1	0.001–0.5	0.001–0.1
*r* ^2^		0.9999	0.9997	0.9992
Repeatability of signals (RSD, %, *n* = 6 dependent injections, and *C* = 0.001 mg/L)		1.89	2.04	2.53
LOD (mg/kg)		0.001	0.0001	0.0005
LOQ (mg/kg)		0.01	0.001	0.005
Recovery (mean ± RSD, %, *n* = 6)	0.01^a^	86.6 ± 5.6	84.1 ± 9.9	108.7 ± 6.1
	0.2^a^	105.7 ± 1.0	83.2 ± 9.9	92.7 ± 7.2
	0.5^a^	96.0 ± 3.2	91.9 ± 7.3	102.7 ± 1.8

^a^Added level, mg/kg.

**Table 4:** Partial validation parameters of triadimenol added in different blank samples.

Parameters		Pollen	Honey	Jelly
Linear equation		*y* = 1.1*∗*10^7^*x* − 3556	*y* = 0.2*∗*10^7^*x* + 6180	*y* = 1*∗*10^7^*x* − 1873
Linear range (mg/L)		0.001–0.1	0.001–0.5	0.001–0.1
*r* ^2^		0.9999	0.9996	0.9996
Repeatability of signals (RSD, %, *n* = 6 dependent injections, and *C* = 0.001 mg/L)		3.34	2.89	2.88
LOD (mg/kg)		0.001	0.0001	0.0005
LOQ (mg/kg)		0.01	0.001	0.005
Recovery (mean ± RSD, %, *n* = 6)	0.01^a^	104.6 ± 6.6	107.3 ± 9.3	106.3 ± 4.1
	0.2^a^	108.4 ± 5.8	106.1 ± 1.8	107.4 ± 7.1
	0.5^a^	106.2 ± 4.0	100.1 ± 5.6	105.1 ± 9.2

^a^Added level, mg/kg.
